# Controlling Gold-Assisted Exfoliation of Large-Area MoS_2_ Monolayers with External Pressure

**DOI:** 10.3390/nano14171418

**Published:** 2024-08-30

**Authors:** Sikai Chen, Bingrui Li, Chaoqi Dai, Lemei Zhu, Yan Shen, Fei Liu, Shaozhi Deng, Fangfei Ming

**Affiliations:** State Key Laboratory of Optoelectronic Materials and Technologies, School of Electronics and Information Technology, Guangdong Province Key Laboratory of Display Material, Sun Yat-sen University, Guangzhou 510275, China; chensk5@mail2.sysu.edu.cn (S.C.); libr7@mail2.sysu.edu.cn (B.L.); daichq5@mail2.sysu.edu.cn (C.D.); zhulm6@mail2.sysu.edu.cn (L.Z.); shenyan7@mail.sysu.edu.cn (Y.S.); liufei@mail.sysu.edu.cn (F.L.); stsdsz@mail.sysu.edu.cn (S.D.)

**Keywords:** mechanical exfoliation, gold-assisted, MoS_2_, transition metal dichalcogenides, pressure

## Abstract

Gold-assisted exfoliation can fabricate centimeter- or larger-sized monolayers of van der Waals (vdW) semiconductors, which is desirable for their applications in electronic and optoelectronic devices. However, there is still a lack of control over the exfoliation processes and a limited understanding of the atomic-scale mechanisms. Here, we tune the MoS_2_-Au interface using controlled external pressure and reveal two atomic-scale prerequisites for successfully producing large-area monolayers of MoS_2_. The first is the formation of strong MoS_2_-Au interactions to anchor the top MoS_2_ monolayer to the Au surface. The second is the integrity of the covalent network of the monolayer, as the majority of the monolayer is non-anchored and relies on the covalent network to be exfoliated from the bulk MoS_2_. Applying pressure or using smoother Au films increases the MoS_2_-Au interaction, but may cause the covalent network of the MoS_2_ monolayer to break due to excessive lateral strain, resulting in nearly zero exfoliation yield. Scanning tunneling microscopy measurements of the MoS_2_ monolayer-covered Au show that even the smallest atomic-scale imperfections can disrupt the MoS_2_-Au interaction. These findings can be used to develop new strategies for fabricating vdW monolayers through metal-assisted exfoliation, such as in cases involving patterned or non-uniform surfaces.

## 1. Introduction

Transition metal chalcogenides (TMDCs), such as MoS_2_, have van der Waals (vdW)-type layered structures. Most of them are semiconductors and possess various intriguing electronic and optical properties, rendering them potential candidates for realizing complex stacked structures and device functions [1,2,3]. Various strategies have been developed to obtain few-layer or single-layer TMDC materials, each with its own advantages and disadvantages. For example, the traditional tape exfoliation method can produce high-quality crystals, but they often suffer from low yield and difficulties in control [4,5]. Liquid-phase and electrochemical exfoliation methods achieve high yields, but typically produce small sizes and can be contaminated by the solution [6,7,8]. Chemical vapor deposition and molecular beam epitaxy methods can produce large-area thin layers, but the crystal quality is strongly affected by the preparation process [9,10]. Given that MoS_2_ is a promising candidate to replace silicon in large-scale integrated circuits, significant effort has been invested in obtaining high-quality monolayers or thin layers with millimeter or even wafer-sized dimensions [11,12,13,14,15].

Recently, it has been discovered that when Au thin films are in contact with TMDCs, large-area and uniform monolayers can be achieved. This process is known as “Au-assisted exfoliation” or “Au-mediated exfoliation” [16,17,18,19,20,21,22,23,24,25,26,27,28,29,30]. The driving force is the strong dispersive interactions between the Au surface and the chalcogen atom plane, often referred to as “covalent-like quasi-bonding” or “strong vdW interactions” [19,22]. The noble metal Au has low chemical activity and remains unoxidized and uncontaminated for several minutes after being exposed to air, making it preferable over other noble metals [22,26,27]. For example, millimeter-scale monolayers are routinely achieved with commercially purchased TMDC crystals, such as MoS_2_ and WSe_2_, with the size and quality of monolayers primarily limited by the bulk crystals [16,19,23,25]. Moreover, the absence of chemical solvents and the mild physical contact ensures high-quality interfaces. For example, ideal metal–semiconductor Schottky contacts and low contact resistance are realized in such metal–TMDC interfaces [31,32]. The monolayers can be transferred to insulating substrates to fabricate electronic devices or stacked to fabricate heterostructures; however, this requires extra etching procedures to remove the Au or to pattern the exfoliated MoS_2_ [23,33]. Exfoliation with patterned Au films has been developed to reduce the etching procedures, such as for obtaining patterned MoS_2_ patches or for exfoliating MoS_2_ directly onto insulating regions [34,35,36,37,38,39]. When a patterned Au surface is used, the key is having a flat surface before forming contacts with the bulk MoS_2_. However, exfoliation yield and reliability still need improvement.

Although it is now clear that the strong vdW interactions are the driving force for exfoliating the top monolayer from bulk TMDCs [16,19], a knowledge gap remains between atomic-scale interactions and macroscale monolayer yield. Specifically, how much of the interface forms such strong vdW interactions and whether other factors are essential for a high exfoliation yield. A major challenge in addressing these questions is that atomic-scale information is concealed within the interface and lost after exfoliation [40,41]. In exfoliation experiments, pressing the contact interface with fingers or soft objects before separating the bulk TMDCs from the metal surface is a common practice [4,16,19,42]. However, a systematic study on the effect of external pressure on Au-assisted exfoliation has not yet been conducted. Intuitively, external pressure should improve the contact and thereby increase the yield. Moreover, controlled tuning of the contact condition through external pressure is expected to provide new knowledge hidden at the interface.

In this work, we conducted a systematic study on the Au-assisted exfoliation of monolayer MoS_2_ with controlled external pressure (“pressure” for short). The exfoliation yield is found to be strongly modulated by pressure. As expected, mild pressure improves the contact between the Au film and MoS_2_, leading to higher yields. Excessive pressure, surprisingly, disrupts the covalent network of MoS_2_ at the interface, resulting in nearly zero exfoliation yield. The loss of exfoliation capability with increased pressure suggests that only a small fraction of MoS_2_ at the interface forms strong vdW interactions with the Au, and the integrity of a continuous MoS_2_ covalent network is also necessary for obtaining large-area monolayers. Scanning tunneling microscopy (STM) measurements of the monolayer MoS_2_/Au interface confirm that even the single-atom-sized imperfections can disrupt the formation of strong MoS_2_-Au vdW interactions. Our results indicate that, although near-unity exfoliation yield can be achieved with Au-assisted exfoliation, there is still significant room for improvement in achieving better interfacial contacts, which is crucial for specific monolayer TMDCs fabrication processes that aim to improve exfoliation yields.

## 2. Materials and Methods

**Au-assisted exfoliation of monolayer MoS_2_ was carried out as follows.** Si slices of 10 × 10 mm^2^ with 300 nm SiO_2_ were used as the substrate for most experiments. After cleaning, a ~2 nm Ti adhesive layer was applied, followed by a coat of Au film to a certain thickness in a magnetron sputtering system. The thicknesses of the Ti and Au layers were calibrated by AFM. Adhesive tape was used to cleave a MoS_2_ crystal chunk (from Taizhou SUNANO New Energy, Shanghai, China) with a lateral size of ~10 mm. The thin sheets left on the tape were further thinned a few more times before being used as the source of MoS_2_. After the Au film was exposed to air, the MoS_2_ side of the tape was quickly placed onto the wafer. The desired pressure was applied and maintained for 2 min before slowly cleaving the tape at an angle of ~45°. Each Au film was used once. The pressure was controlled by a translation stage (no force gauge). The exfoliation process was performed in a cleanroom with a temperature of 25 °C and a humidity of 45%. A Au(111)/mica sample (200 nm Au; from PrMat, Shanghai, China) was also used as a substrate. It was fixed on a flag-type sample holder and cleaned by Ar ion sputtering and ~500 °C annealing in an ultrahigh vacuum environment. Due to the weak adhesion between the Au film and the mica, the Au(111)/mica sample remained fixed in the sample holder during the exfoliation procedure. The exfoliation was either performed in air with hand-applied pressure (the low-pressure case) or in situ within the vacuum system on a translation stage without a pressure gauge (the high-pressure case). For experiments where the pressure was applied by hand, the back side of the tape was pressed with a soft cloth held by hand for two minutes.

**Characterization of the Au surface with exfoliated MoS_2_ was carried out as follows.** Optical microscopes (Zeiss LSM700 and AxioScope AI confocal microscope, Oberkochen, Germany) were used to locate the exfoliated MoS_2_ on the Au surface. Three separate 1 × 1 mm^2^ square regions with the highest MoS_2_ coverage on the same sample were selected for characterizing the exfoliation yield (*Y*) and monolayer fraction (*F*_1L_). Fiji/ImageJ software (version no. 1.54f) was used to select the desired regions in the square images. Raman spectroscopy was performed using a RENISHAW inVia Raman system (London, UK) using a 532 nm laser at a power of 0.6 mW. AFM was performed using a Bruker Dimension Fastscan (Billerica, Massachusetts, MA, USA) with the peak force tapping mode in an ambient atmosphere. STM measurements were carried out using a variable temperature STM (Omicron, Taunusstein, Germany) operating in an ultra-high vacuum (base pressure higher than 1 × 10^−9^ mbar) at room temperature. Point d*I*/d*V* spectra were obtained by differentiating the smoothed *I*(*V*) curves.

## 3. Results and Discussion

**Figure 1:**
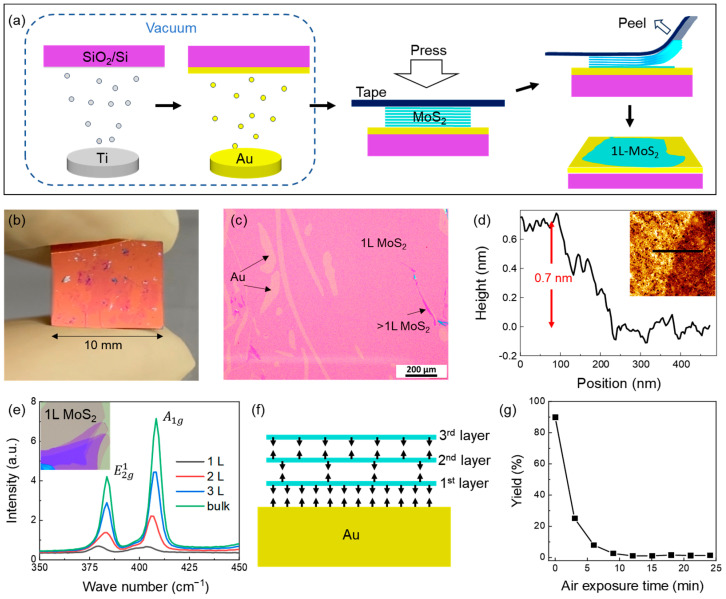
The procedure and results of Au-assisted exfoliation of large-area 1L-MoS_2_. (**a**) Schematic representation of the exfoliation process: preparing the Au film on a slice of Si wafer using a magnetron sputtering system operating in vacuum; contacting the Au film with flakes of bulk MoS_2_ attached to the tape in air; peeling the tape and obtaining 1L-MoS_2_ on the Au film. (**b**) A photo of the sample. (**c**) An optical microscope image of the exfoliated sample. Most of the surface areas were covered with 1L-MoS_2_. A small amount of multilayer MoS_2_ and exposed Au regions are labeled. (**d**) A line profile extracted from the AFM image (inset) of the 1L-MoS_2_ boundary. (**e**) Raman spectra of MoS_2_ with different thicknesses. (**f**) A sketch of the interfacial forces between the Au film and the top few layers of MoS_2_. The arrows represent the inter-layer forces. (**g**) The yield of MoS_2_ as a function of the Au film’s air exposure time.

Figure 1a shows the schematic of the operation process of the Au-assisted exfoliation of the monolayer MoS_2_. A clean 10 mm × 10 mm SiO_2_/Si wafer was used as the substrate and loaded into the vacuum chamber to prepare the Au film. A 2 nm Ti layer was deposited as the adhesion layer before depositing an 18 nm Au film. After being removed from vacuum, the sample was brought into contact with thinned MoS_2_ bulk flakes attached to the tape as quickly as possible. After gently pressing the tape with a soft cloth for two minutes, one side of the tape was slowly peeled off at an angle of about 45° from the substrate. Large-area and uniform monolayers MoS_2_ (1L-MoS_2_) could be identified by the naked eye (a photo of the sample is shown in Figure 1b). In an optical microscope image, as shown in Figure 1c, the surface was mostly covered with continuous 1L-MoS_2_. Atomic force microscopy (AFM) measurements of the film and its boundary show that it had a thickness of ~0.7 nm (Figure 1d), consistent with its monolayer thickness. The Raman spectroscopy results of the single-layer and multi-layer regions (Figure 1e) show the characteristic peaks of MoS_2_ at around 384 cm^−1^ and 408 cm^−1^, which significantly decrease when the thickness decreases, consistent with previous reports [42,43]. A microscopic physical illustration of the strong vdW interactions between the MoS_2_ and Au within the interface is shown in Figure 1f. The first layer of MoS_2_ was adsorbed onto the Au surface, forming strong vdW interactions. Meanwhile, the lattice mismatch between the MoS_2_ and Au weakened the vdW force between the first and the second layers of MoS_2_, which is the weakest interface of the system. The Au film may be contaminated and lose its exfoliation capability if exposed to air for a prolonged period of time before contacting the bulk MoS_2_. As shown in Figure 1g, the yield quickly dropped to nearly zero after more than 10 min of air exposure. All later described experiments were conducted by forming the MoS_2_-Au contacts within 1 min after removing the Au film from the vacuum.

**Figure 2:**
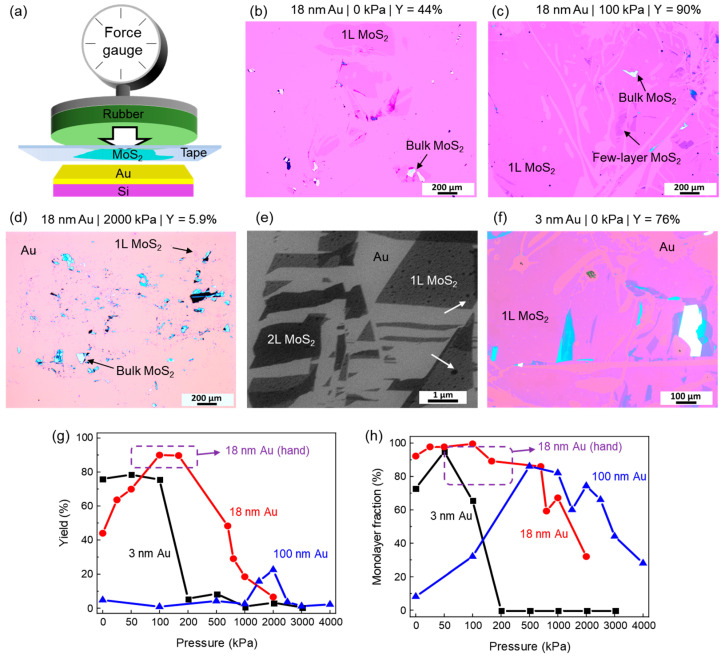
Au-assisted exfoliation of 1L-MoS_2_ under controllable external pressure. (**a**) Schematic diagram of the pressing device with a pressure sensor. (**b**–**d**) Typical optical microscope images of the 18 nm Au sample after exfoliation. The thickness of the Au films, the pressure (*P*), and the yield (*Y*) are labeled at the top of each image. The surface features, including the 1L-MoS_2_, uncovered Au, and bulk MoS_2_ fragments, are labeled. (**e**) SEM image of the surface of the 18 nm Au sample after exfoliation with *P* = 2000 kPa. Regions with MoS_2_ and bare Au are labeled. Several small bubbles within the MoS_2_ regions are indicated by arrows. (**f**) An optical microscope image similar to (**b**–**d**), but the sample is made with a 3 nm Au film. (**g**) Statistical values of yield (*Y*) as a function of pressure. The plot shows three sets of data obtained using 3 nm, 18 nm, and 100 nm Au films, respectively. (**h**) Statistical values of the monolayer fraction (*F*_1L_) obtained from the same set of samples as in (**g**). The dashed-line boxes in (**g**,**h**) mark the parameter ranges obtained from hand-made samples. The *x*-axes in (**g**,**h**) are non-linear for clarity.

To systematically study the effect of pressure on the exfoliation yield, a translation stage with a force sensor was used to apply a constant vertical force for two minutes during the contact between the bulk MoS_2_ and Au film. The schematic diagram of the device is shown in Figure 2a, and its photo is shown in Figure S1. After the Au film on a 10 mm × 10 mm Si wafer was placed on the lower stage, the bulk MoS_2_ flakes attached to a piece of tape were placed on top to form a contact (the pressure was recorded as 0). The upper plate, covered by a soft 2 mm rubber gasket, exerted external pressure to the tape from the backside. The rubber gasket was larger than the wafer. By assuming a uniform distribution of the exerted force, the force reading was converted into the external vertical pressure (*P*), which was the main control parameter for the experiments. Other sources of vertical pressure, such as the elastic force of the tape and gravity, were relatively small and remained unchanged, and were, therefore, omitted from our study.

Several typical optical microscope images of the surfaces of the 18 nm Au films after exfoliating the MoS_2_ are shown in Figure 2b–d; more are shown in Figure S2. The statistical results of the samples are summarized in Figure 2g,h, which display the pressure-dependent yield (*Y*) and monolayer fraction (*F*_1L_), respectively. These statistics are calculated based on three separate 1 mm × 1 mm-sized optical microscope images (*S_fov_*), with the highest MoS_2_ coverage on the sample surface: the total area covered by MoS_2_ was recorded as *S_all_* and the total area covered by the 1L-MoS_2_ was *S*_1L_; they resulted in *Y* =$\sum_{k=1}^{3}\left(S_{all}^{k}/S_{fov}^{k}\right)/3\times 100\%$, and *F*_1L_$=\sum_{k=1}^{3}\left(S_{1L}^{k}/S_{all}^{k}\right)/3\times 100\%$. The regions of 1L-MoS_2_ were identified by their contrast with the clean Au surface in the optical microscope images. The statistical results from multiple sets of repeated measurements using the same pressure indicate that the errors in *Y* and *F*_1L_ are within 16%, as shown in Figure S3. Since the MoS_2_ exfoliation yield was not uniform across the whole 10 mm × 10 mm Si wafer, in order to reflect the formation of the millimeter-sized 1L-MoS_2_ regions, we chose a *S_fov_* of 1 mm × 1 mm for the statistics (see more detailed explanations in the caption of Figure S3). The obtained statistical values of *Y* and *F*_1L_ depend on the size of the *S_fov_*, because fewer 1L-MoS_2_ covered regions are involved in the statistics for larger *S_fov_*. For instance, using images with *S_fov_* = 2 mm × 2 mm for the statistics would result in a lower yield. Nevertheless, the overall behavior and our conclusions do not depend on the choice of *S_fov_*, as shown in Figure S3.

The yield on an 18 nm Au film at zero pressure was 44% (Figure 2b), while it increased to 90% at around 100 kPa (Figure 2c). The yields at these pressures were almost solely composed of 1L-MoS_2_, or *F*_1L_ ≈ 100% (Figure 2h), indicating a successful production of large-area 1L-MoS_2_ with the translation stage. As a consistency check, six samples prepared with hand-applied pressures were analyzed, as shown in Figure S4. The data indicate a *Y* range from 82% to 95% and a *F*_1L_ range from 75% to 97%. The hand-applied pressure was estimated to be between 50 and 300 kPa. The ranges are marked by dashed boxes in Figure 2g,h, showing consistent results with those obtained using the translation stage, which, however, provided quantitative control as well as a wider range in pressure. With higher pressure at *P* > 200 kPa, the yield dropped steeply, as shown in Figure 2d,g. At *P* = 2000 kPa, the yield primarily consisted of small pieces of multi-layer MoS_2_, as seen in Figure 2d and the significant drop of *F*_1L_ in Figure 2h. In addition to these fragments with a ~10 µm size, as seen in the optical microscope images, smaller-sized fragments were also widely distributed on the surface and could be resolved in SEM images, as shown in Figure 2e. These nanometer-sized fragments of MoS_2_ show darker contrast and have straight boundaries. Small bubbles appeared in the MoS_2_-covered region (indicated by arrows), which distinguishes the MoS_2_ from the bare Au surface. 

The pressure-dependent exfoliation experiments were repeated using 3 nm and 100 nm Au films. One representative image is shown in Figure 2f, with complete sets provided in Figure S2. The 3 nm Au film resulted in *Y* = 76% at zero pressure (Figure 2f,g), higher than that of the 18 nm Au film. However, increasing the pressure initially did not significantly enhance *Y*, which rapidly dropped below 10% after *P* > 100 kPa (Figure 2g), accompanied by a sharp decrease in *F*_1L_ (Figure 2h). For the 100 nm Au film, the yield remained low at about 5%, increase to approximately 20% at *P* ≈ 2000 kPa, and decreased again to 5% at higher *P*. Overall, the pressure-dependent yields from the three Au films with different thicknesses in Figure 2g show peak-like characteristics, though the peak center for the 3 nm Au film may be near *P* = 0. The left side of the “peak” represents a pressure-assisted exfoliation, such as the *P* = 0 to 100 kPa range for the 18 nm Au film, which is consistent with the intuitive idea that external pressure improves contact and facilitates exfoliation. To test this hypothesis, the surfaces of the 3 nm, 18 nm, and 100 nm Au films are characterized by AFM before and after the exfoliation experiment at *P* = 1000 kPa (in areas not covered by MoS_2_), as shown in Figure S4. The surface roughness values were 0.37 nm, 0.38 nm, and 1.2 nm, respectively, before applying pressure; these values consistently decreased to 0.28 nm, 0.30 nm, and 0.73 nm, respectively, after the pressure. The decrease in roughness was likely due to the deformation of atomic-scale grains in the Au films, and the resulting smoother surface would have promoted better contact between the MoS_2_ and the Au film. Notably, the 100 nm Au film exhibited significantly larger roughness than the 3 nm and 18 nm Au films, and its best yield remained low, consistent with previous reports [16].

**Figure 3:**
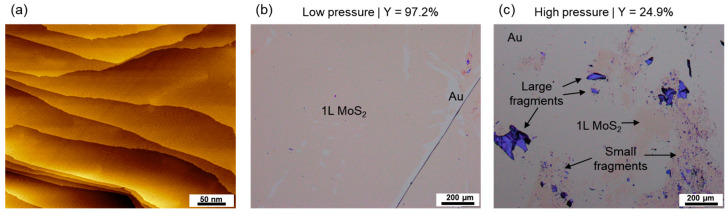
Exfoliation experiment using an ultra-flat Au(111) film. (**a**) STM images of a 200 nm Au(111) film after cleaning and annealing in an ultra-high vacuum, showing atomic terraces and steps. Optical microscope images of the Au surface after exfoliation with low pressure (**b**) and with excessive pressure (**c**). The *Y* values for (**b**,**c**) are labeled above their images.

A surprising result shown in Figure 2g is that *Y* dropped to ~5% at high *P*, such as for the data points with *P* ≥ 200 kPa for 3 nm Au or *P* ≥ 2000 kPa for 18 nm Au. This means that the Au films lost their exfoliation capability once present at lower pressures. To check whether this behavior persisted when using a smoother Au film, a vacuum-cleaned, crystallized Au(111) film was used to perform the exfoliation experiment, as presented in Figure 3. The STM image of the as-prepared Au substrate (Figure 3a) shows that the surface was atomically flat with wide terraces and atomic steps with a height of ~0.24 nm. After exfoliating the MoS_2_ with a hand-applied external pressure (indicated as “low pressure”), the surface was uniformly covered with 1L-MoS_2_, with only a few bare Au regions, as shown in Figure 3b. The statistics show a *Y* of 97.2% and an *F*_1L_ close to 100%, which represents the best exfoliation result in our experiment. When applying much higher pressure (estimated to be 1000 kPa), *Y* dropped significantly to 24.9%, and the residue on the surface was composed of a significant number of multilayer MoS_2_ fragments, as shown in Figure 3c. Notably, the exfoliation at high pressure was performed in situ within the ultra-high vacuum environment, ensuring that the surface of the Au film was almost entirely free of contaminants. These results confirm that the loss of exfoliation capability under excessive pressure is universal and not related to the surface roughness or gas adsorption.

The loss of the exfoliation capability is an unexpected behavior, as the additional pressure should have increased or at least not decreased the contact area between the bulk MoS_2_ and the Au film at the interface. One would expect *Y* to increase or at least remain unchanged if one assumed that the contact area in the interface roughly corresponds to the area for the MoS_2_ exfoliated onto the Au film. However, this assumption must be incorrect. The fact that *Y* consistently dropped to ~5% at excessive pressures suggests that only a small fraction of the MoS_2_-Au interface formed strong vdW interactions, which enabled the MoS_2_ to be exfoliated from its bulk. Here, the figure of 5% could be taken as an estimate of the upper limit of such a fraction. The majority of the MoS_2_ at the interface (e.g., >95%) does not form strong vdW interactions and is expected to remain attached to the bulk MoS_2_ before exfoliation. Obtaining large-area 1L-MoS_2_ with high *Y* relies on the exfoliation of these unbonded regions under specific physical conditions, which can be disrupted by excessive pressure. It is worth noting that the drop in *Y* at excessive pressure was always accompanied by a presence a large number of multilayer MoS_2_ fragments left on the Au surface, as shown in Figure 2d and Figure 3c and Figure S2. These micrometer- or nanometer-sized MoS_2_ fragments indicate that the in-plane covalent bond network of the MoS_2_ film had broken under excessive pressure. The majority of the nonbonded MoS_2_ at the interface would not exfoliate toward the Au surface if it was no longer connected to the bonded regions through these covalent networks. Upon peeling the tape, only the MoS_2_ fragments with strong vdW interactions with the Au surface (<5%) remained on the Au surface, resulting in a low yield and a large number of fragments.

**Figure 4:**
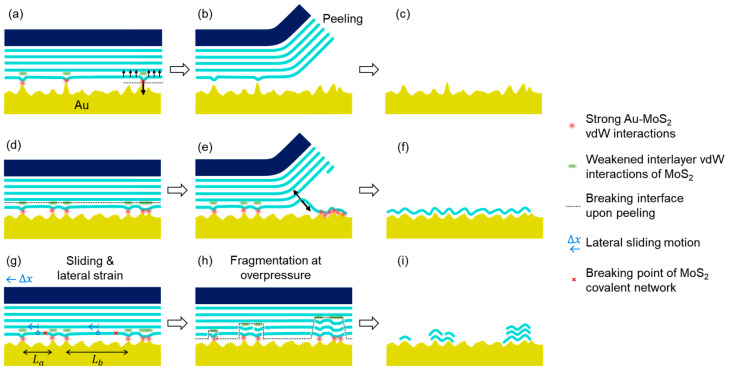
Schematic diagram of the pressure-controlled interface and exfoliation process. The exfoliation yields were low when the density of the MoS_2_-Au interactions was insufficient (**a**–**c**), or when the covalent network of the MoS_2_ layers within the interface broke (**g**–**i**). However, a high yield of large-area monolayers was obtained when the density of the MoS_2_-Au interactions was sufficiently high and the covalent network remained intact (**d**–**f**). The arrows in panels (**a**,**e**) represent the competing forces acting on the local regions of the first MoS_2_ layer. The shapes and orientations of the arrows between the panels indicate the sequence of the experiment.

We summarize the atomic-scale interactions and key physical mechanisms related to the pressure-controlled exfoliation in Figure 4. When the bulk MoS_2_ was brought into contact with the Au film, a small fraction of the MoS_2_ surface formed strong vdW interactions (highlighted with red dots), which concurrently weakened the vdW interactions between the first and second MoS_2_ layers (highlighted with green bars), as shown in Figure 4a. The remaining regions were nonbonded to the Au surface due to the atomic-scale corrugations at the interface. Whether the first-layer MoS_2_ remained on the Au surface or not upon peeling was determined by the competition between the strong MoS_2_-Au vdW interactions and the interlayer vdW interactions within the MoS_2_ (arrows shown in Figure 4a). If the strong vdW interactions were insufficient in strength or density, e.g., when the Au surface was rough, the first layer of MoS_2_ would detach from the Au surface, resulting in low yield (Figure 4b,c). Better MoS_2_-Au contact could be achieved by using a flatter Au surface or by applying vertical pressure, which induced structural deformations of the nano-grains of the Au film (Figure 4d). The strong vdW interactions facilitate the attachment of local MoS_2_ regions toward the Au surface. The interaction can be further enhanced when the first MoS_2_ layer detaches from the bulk MoS_2_ due to its flexible two-dimensional character, allowing these MoS_2_ regions to anchor to the Au surface. During the peeling of the tape, the nonbonded regions of the first MoS_2_ layer were pulled toward the Au surface through the complete covalent network, resulting in a continuous large-area 1L-MoS_2_ on the Au surface (Figure 4e,f).

In addition to the strong vdW interactions at the MoS_2_-Au contacts, the integrity of the covalent network of the first MoS_2_ layer is a prerequisite for the successful exfoliation of large-area 1L-MoS_2_. The yield dropped to nearly zero when the integrity of the MoS_2_ was destroyed at excessive pressures. The fragmentation of the MoS_2_ indicates the presence of overwhelming in-plane stretching forces within the interface. Such in-plane forces could be induced by the microscale deformation of the tape or the lateral motion of the pressing equipment, as well as the deformation or cracking of the Au film, all of which were likely positively correlated to the external vertical pressure. When the bulk MoS_2_ slid laterally relative to the Au surface, a strong stretching force built up within the first-layer MoS_2_ (e.g., the regions indicated by red crosses in Figure 4g), as several regions of MoS_2_ followed the motion of the bulk MoS_2_, while others were anchored by the Au surface and stayed intact. The covalent network of the MoS_2_ can break at excessive stretching strain. It is important to note that the first-layer MoS_2_ forming strong vdW interactions with the Au comprised only a small fraction of the interface (<5%, as presented previously); the exfoliation capability would be lost if the rest of the non-bonded MoS_2_ regions could not be exfoliated due to the breaking of the covalent network. The noticeable presence of multilayer MoS_2_ can be explained by the covalent network breaking at deeper layers in the bulk MoS_2_.

This physical picture is also qualitatively consistent with the more subtle correlation of pressure and Au film thickness-dependent behaviors. Specifically, the fragmentation of the MoS_2_ appeared to occur at higher pressures when the Au film was thicker. For example, the pressure where *Y* and *F*_1L_ dropped significantly was 100~200 kPa for 3 nm Au, 500~1000 kPa for 18 nm Au, and 2000~3000 kPa for 100 nm Au, as shown in Figure 2g,h. This behavior can be rationalized by the simple one-dimensional model shown in Figure 4g: Assume the Au-bonded regions (indicated by the red dots) are stationary, while the center of the non-bonded regions (indicated by the blue circles) follows the bulk MoS_2_ to slide by ∆*x*. The stretching strain (at the red crosses) is proportional to 2∆*x*/*L*, where *L* is the length of the non-bonded MoS_2_ region. As ∆*x* increases, MoS_2_ regions with a smaller *L* will reach the breaking point first; for example, the critical strain for the breaking of the covalent bonds in the “*L_a_*” region is reached at a smaller ∆*x* value than in “*L_b_*”. The average size of the non-bonded MoS_2_ regions (represented by *L* in the model) within the interface would be smaller for flatter, or equivalently thinner, Au films (see Figure S5), and therefore, their covalent network would break at a lower *P*. In our experiment, the absence of an increase in the exfoliation yield for the 3 nm Au film and its lower yield at mild pressures, e.g., 100~200 kPa, can be explained by the early fragmentation of MoS_2_, as shown in Figure 2g. On the other hand, the late fragmentation of MoS_2_ for the 100 nm Au possibly made the pressure-induced increase in yield appear at much higher pressures, e.g., near 1000~2000 kPa, while the subsequent decrease in yield could also be attributed to the pressure-induced fragmentation of the MoS_2_. Moreover, it has been shown that rougher Au surfaces enhance the in-plane strain of the MoS_2_ monolayer [44], which could possibly result in the formation of fragments on thicker Au films at a low *P*, as shown in Figure 2h, where the 100 nm Au film shows low *F*_1L_ values at a low *P* as opposed to the other two thinner Au films.

**Figure 5:**
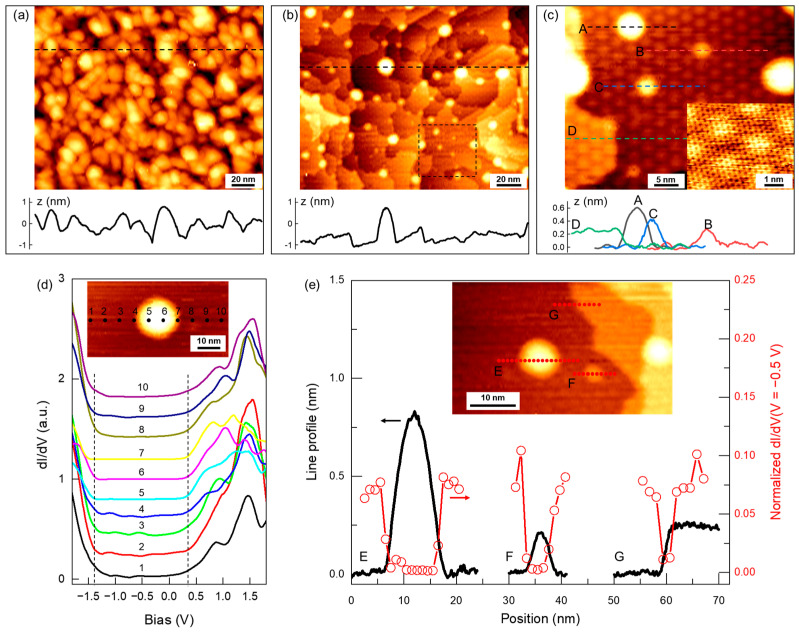
STM characterization of the Au surface covered with 1L-MoS_2_. (**a**) STM image (*V*_*b*_ = 2 V; *I*_*t*_ = 0.05 nA) of the as-prepared 1L-MoS_2_/Au sample. (**b**) STM image (*V*_*b*_ = 2 V; *I*_*t*_ = 0.05 nA) of the sample after annealing at 350 °C. (**c**) Small-scale STM image (*V*_*b*_ = 1 V; *I*_*t*_ = 0.05 nA) of the area indicated by a dashed square in (**b**). The inset is a high-resolution image (*V*_*b*_ = 1 V; *I*_*t*_ = 0.3 nA) showing the MoS_2_ lattice. The lower parts of the (**a**–**c**) panels show the line profiles marked by the horizontal dashed line in the figures, and they share the same horizontal scale with their corresponding images. Four types of line profiles with different structures are displayed in (**c**), where “A”, “B”, and “C” are all different sizes of bubble-like structures, and “D” is an atomic step of Au(111). (**d**) *dI*/*dV* spectra taken at positions along a line crossing one bubble, as indicated by the numbers shown in the inset image. The vertical dashed lines mark the apparent semiconducting gap regions. (**e**) Overlay of line profiles (black curve) and normalized *dI*/*dV*(*V* = −0.5 V) as a function of position (red line with circles) for three different surface structures shown in the inset. “E” and “F” label one large and one small bubble-like surface structure, respectively, while “G” labels a Au(111) step.

To better understand the formation of strong vdW interactions between the Au film and MoS_2_, the surface of an 18 nm Au film covered with 1L-MoS_2_ was characterized by STM with atomic-scale resolution. Figure 5a shows a large-scale STM image of the as-prepared sample, where the surface appears to be composed of densely packed particles with lateral dimensions of 10–20 nm and apparent heights of 1~2 nm. The morphology reflects the structure of the underlying amorphous Au film. However, detailed characterization by STM is challenging due to significant surface corrugation and residual gas molecules. The sample was then annealed at 350 °C in an ultra-high vacuum to crystallize the Au film and degas the sample. Further STM measurements were performed in situ without leaving the vacuum. Figure 5b shows that the surface after annealing was composed of atomic terraces with lateral dimensions ranging from 10 to 50 nm, along with atomic steps typically showing a height of ~0.24 nm, consistent with the atomic step of the Au(111) lattice. In a smaller-scale STM image shown in Figure 5c, the terraces show triangular lattices with periodicities of 2–3 nm. These are moiré superlattices formed between the Au(111) surface and the monolayer MoS_2_, and the periodicity depends on their relative twisting angles [45,46]. These moiré superlattices covered the entire surface, indicating a uniform and continuous 1L-MoS_2_ layer on top of the Au film. A fine scan showed the MoS_2_ lattice with a lattice constant of ~0.32 nm (inset of Figure 5c) coexisting with the moiré superlattice.

In addition to the terraces and steps, the surface also displayed a significant number of bubble-like features, as seen in Figure 5b. Most of these features had lateral dimensions ranging from 1 to 10 nm and heights ranging from 0.1 to 2 nm. They were likely formed by aggregated molecules trapped at the interface. The d*I*/d*V* spectra (proportional to the density of states (DOS)) along a line crossing a bubble are shown in Figure 5b. The spectra show overall consistent features, e.g., an apparent semiconducting gap from −1.4 V to +0.35 V, consistent with the ~1.8 eV semiconducting gap of monolayer MoS_2_ [47]. Upon closer inspection of the d*I*/d*V* spectra, it can be observed that spectra “5~7” taken on the bubble have zero DOS within the gap region, while other spectra taken on flat terraces show small but non-zero DOS within the gap. Such in-gap DOS indicates that the MoS_2_ acquired a metallic character through band hybridization with the Au surface, while the interstitial molecules effectively decoupled the MoS_2_ and Au, allowing the MoS_2_ to regain its intrinsic semiconducting character. These in-gap states indicate the formation of strong vdW interactions between the MoS_2_ and Au. As shown in Figure 5e, the d*I*/d*V* point spectra were measured at several different types of imperfections in the same image, including two bubbles (labeled as “E” and “F”) and a Au(111) atomic step edge (labeled as “G”). The locations for these spectra are marked by dots in the inset image, while the line profiles crossing these three features are displayed in the plot. The d*I*/d*V* spectra are provided in Figure S6. To highlight the in-gap states, the d*I*/d*V* value at *V* = −0.5 V (near the center of the apparent semiconducting gap) was extracted from each spectrum and normalized to the d*I*/d*V* value at *V* = 1.0 V (in the conduction band). The normalized d*I*/d*V* (*V* = −0.5 V) values at each location are shown in Figure 5e, overlaid with the line profiles. The normalized d*I*/d*V* (*V* = −0.5 V) was approximately 0.075 at positions on the flat terraces. It dropped to nearly zero in the bubble regions (“E” and “F”), as well as the Au(111) atomic step (“G”). These regions with d*I*/d*V* (*V* = −0.5 V) ≈ 0 were well-confined to these imperfections, as evidenced by comparing the d*I*/d*V* (*V* = −0.5 V) curves with the line profiles. Note that the Au(111) step (“G”) and the “F” bubble exhibit heights of ~0.2 nm, which is comparable to the size of single atoms, and they could be considered the lower limit of imperfect interfacial structures at the MoS_2_-Au interface. The formation of strong MoS_2_-Au vdW interactions was disrupted by these smallest imperfections, demonstrating the stringent conditions required to form such strong vdW interactions. This finding aligns with the conclusion that only a small fraction of the MoS_2_-Au interface can form strong vdW interactions.

## 4. Conclusions

In summary, we conducted a comprehensive study on the external pressure effect on the Au-assisted exfoliation of large-area 1L-MoS_2_. The vertical pressure improved the MoS_2_-Au contact and enhanced the yield, whereas accompanying lateral strain within the interface caused the MoS_2_ to break into micro- or nanometer-sized fragments, resulting in a nearly zero yield. Such behaviors indicate two prerequisites for the production of large-area 1L-MoS2. One is the formation of strong MoS_2_-Au vdW interactions with a sufficient density, allowing for the creation of anchoring regions of MoS_2_ on the Au surface; however, these regions always constitute a small fraction of the interface (estimated upper limit: 5%). The other is the exfoliation of the remaining regions through the continuous in-plane covalent network of the first-layer MoS_2_. The external pressure and the surface roughness of the Au film influence both factors, and the best yield is achieved by tuning the parameters to maximize the MoS_2_-Au vdW interactions while avoiding the breaking of the MoS_2_ covalent network. STM studies of different surface structures at the 1L-MoS2/Au interface revealed that even single-atom-sized imperfections can disrupt the formation of strong vdW interactions between MoS_2_ and Au, emphasizing the stringent conditions required for such interactions.

These findings indicate that, despite the high yield currently achievable in metal-assisted exfoliation for large-area monolayer TMDCs, there is still considerable room for improvement. This is particularly important for patterned Au film structures, which inherently have non-flat interfaces and face challenges in both yield and consistency. Moreover, our results are broadly applicable to various types of TMDC and possibly other layered vdW materials and will aid in the development of new exfoliation methods by exploiting the intricate interactions at the interface.

## Data Availability

The data are contained within the article and Supplementary Materials.

## Supplementary Materials

The following supporting information can be downloaded at: https://www.mdpi.com/article/10.3390/nano14171418/s1, Figure S1. Experimental setup for Au-assisted exfoliation of 1L-MoS_2_ with controlled external pressure. Figure S2. Typical optical microscope images of samples obtained after exfoliation under specific external pressure conditions. Figure S3. Consistency check of the statistics of the exfoliation yield. Figure S4. Optical microscope images of six samples made with hand-applied pressures. Figure S5. Atomic force microscopy morphology of gold films both before and after applying pressure. Figure S6. d*I*/d*V* spectra of the imperfections on the 1L-MoS_2_-covered Au surface.
