# Supplementary material for: Controlling Gold-Assisted Exfoliation of Large-Area MoS2 Monolayers with External Pressure
Source: Nanomaterials (Basel). 2024 Aug 30;14(17):1418. doi: 10.3390/nano14171418 (PMC11397389; doi:10.3390/nano14171418)
Supplement: Supplementary file 1 [file nanomaterials-14-01418-s001.zip › nanomaterials-3176086-SI.pdf]

Supplementary Materials

Controlling Gold-Assisted Exfoliation of Large-Area MoS2 Monolayers with External Pressure

Sikai Chen †, Bingrui Li †, Chaoqi Dai, Lemei Zhu, Yan Shen, Fei Liu, Shaozhi Deng and Fangfei Ming *

State Key Laboratory of Optoelectronic Materials and Technologies, School of Electronics and Information Technology, Guangdong Province Key Laboratory of Display Material, Sun Yat-sen University, Guangzhou 510275, China; chensk5@mail2.sysu.edu.cn (S.C.); libr7@mail2.sysu.edu.cn (B.L.); daichq5@mail2.sysu.edu.cn (C.D.); zhulm6@mail2.sysu.edu.cn (L.Z.); shenyan7@mail.sysu.edu.cn (Y.S.); liufei@mail.sysu.edu.cn (F.L.); stsdsz@mail.sysu.edu.cn (S.D.)

* Correspondence: mingff@mail.sysu.edu.cn

† These authors contributed equally to this work.

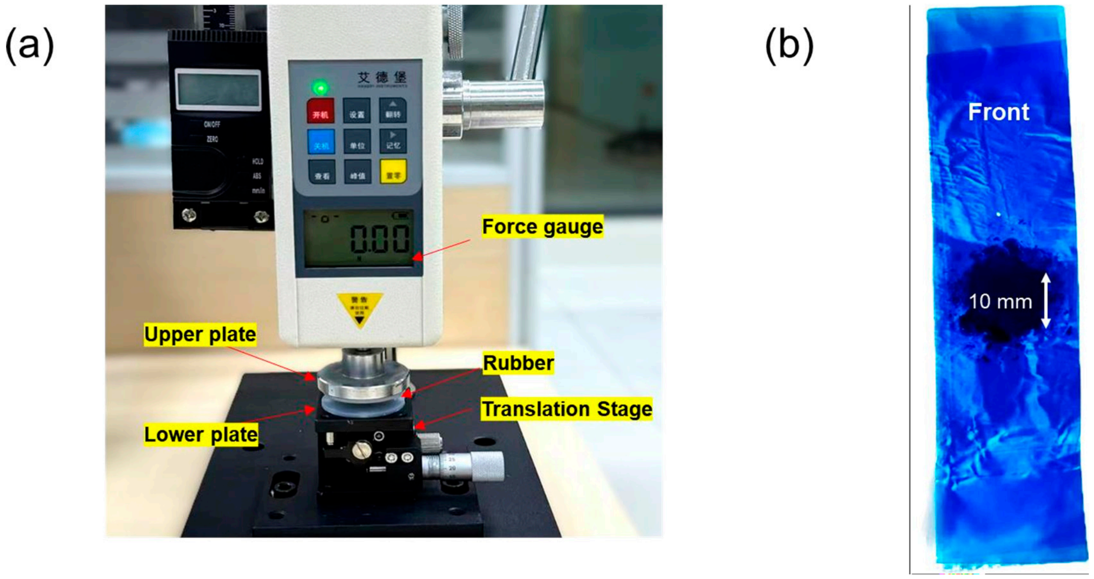

Figure S1. Experimental setup for Au-assisted exfoliation of 1L-MoS_2 with controlled external pressure. (a) Photograph of the pressing device with a pressure sensor. (b) Flakes of bulk MoS_2 attached to tape, which are exfoliated from a piece of thick bulk MoS_2 crystal. To perform the Au-assisted exfoliation, the tape is gently placed onto the prepared Au film on a 10 mm × 10 mm Si wafer, which is positioned on the translation stage with the top facing upwards. The head of the force gauge is covered with 2 mm thick silicone gaskets and is larger than the Si wafer.

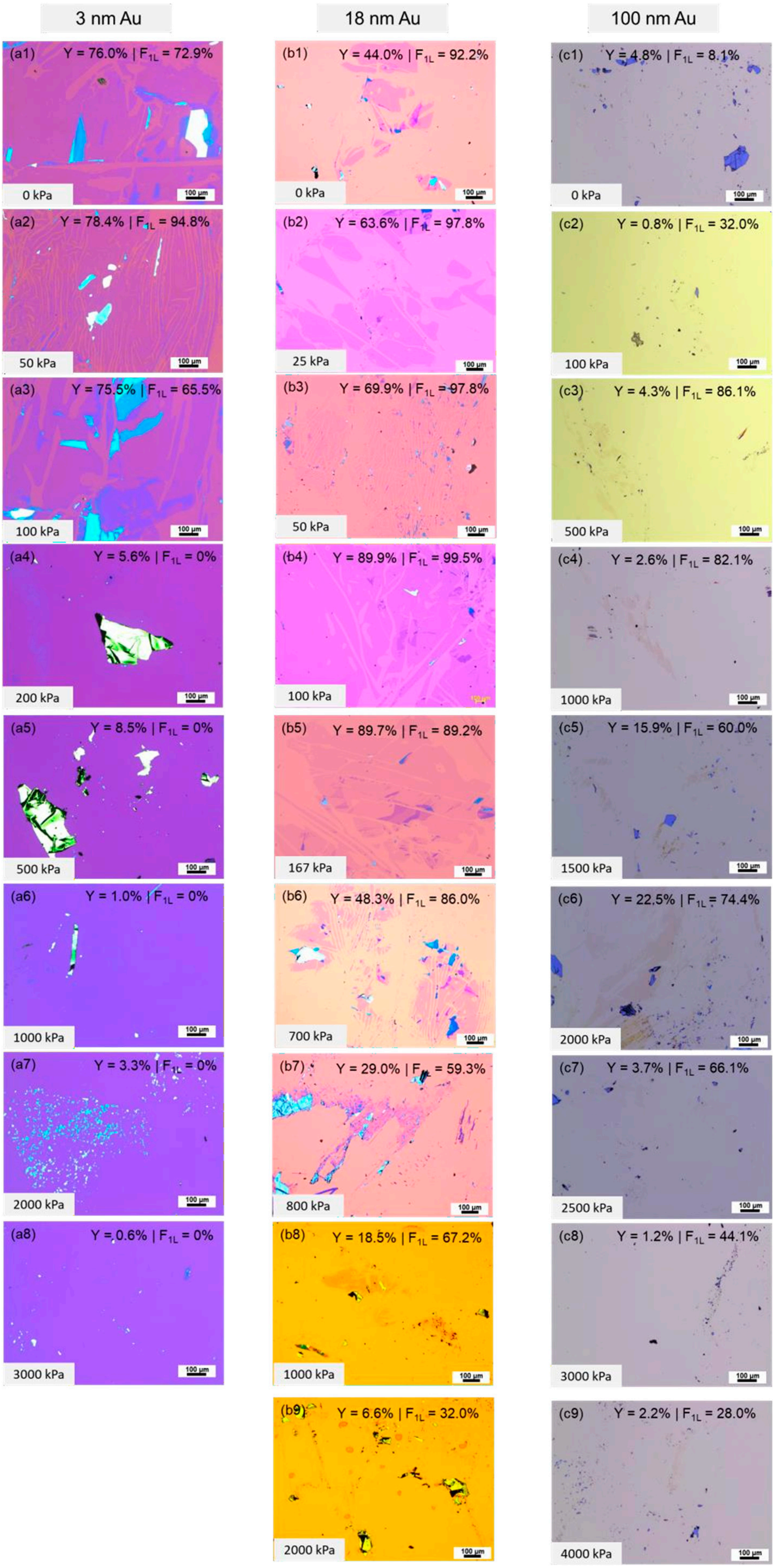

3 nm Au
18 nm Au
100 nm Au
(a1) Y = 76.0% | F1L = 72.9%
0 kPa
(a2) Y = 78.4% | F1L = 94.8%
50 kPa
(a3) Y = 75.5% | F1L = 65.5%
100 kPa
(a4) Y = 5.6% | F1L = 0%
200 kPa
(a5) Y = 8.5% | F1L = 0%
500 kPa
(a6) Y = 1.0% | F1L = 0%
1000 kPa
(a7) Y = 3.3% | F1L = 0%
2000 kPa
(a8) Y = 0.6% | F1L = 0%
3000 kPa
(b1) Y = 44.0% | F1L = 92.2%
0 kPa
(b2) Y = 63.6% | F1L = 97.8%
25 kPa
(b3) Y = 69.9% | F1L = 97.8%
50 kPa
(b4) Y = 89.9% | F1L = 99.5%
100 kPa
(b5) Y = 89.7% | F1L = 89.2%
167 kPa
(b6) Y = 48.3% | F1L = 86.0%
700 kPa
(b7) Y = 29.0% | F = 59.3%
800 kPa
(b8) Y = 18.5% | F1L = 67.2%
1000 kPa
(b9) Y = 6.6% | F1L = 32.0%
2000 kPa
(c1) Y = 4.8% | F1L = 8.1%
0 kPa
(c2) Y = 0.8% | F1L = 32.0%
100 kPa
(c3) Y = 4.3% | F1L = 86.1%
500 kPa
(c4) Y = 2.6% | F1L = 82.1%
1000 kPa
(c5) Y = 15.9% | F1L = 60.0%
1500 kPa
(c6) Y = 22.5% | F1L = 74.4%
2000 kPa
(c7) Y = 3.7% | F1L = 66.1%
2500 kPa
(c8) Y = 1.2% | F1L = 44.1%
3000 kPa
(c9) Y = 2.2% | F1L = 28.0%
4000 kPa
100 µm

Supplementary Figure S2. Typical optical microscope images of samples obtained after exfoliation under specific external pressure conditions. Results from three different sets, using 3 nm, 18 nm, and 100 nm Au films, are shown in three separate columns. The pressure, exfoliation yield (Y), and monolayer fraction (F_{1L}) are labeled in the images. The variations in background colors are due to the use of different microscopes and/or varying light balance conditions, which do not affect the data statistics or analysis.

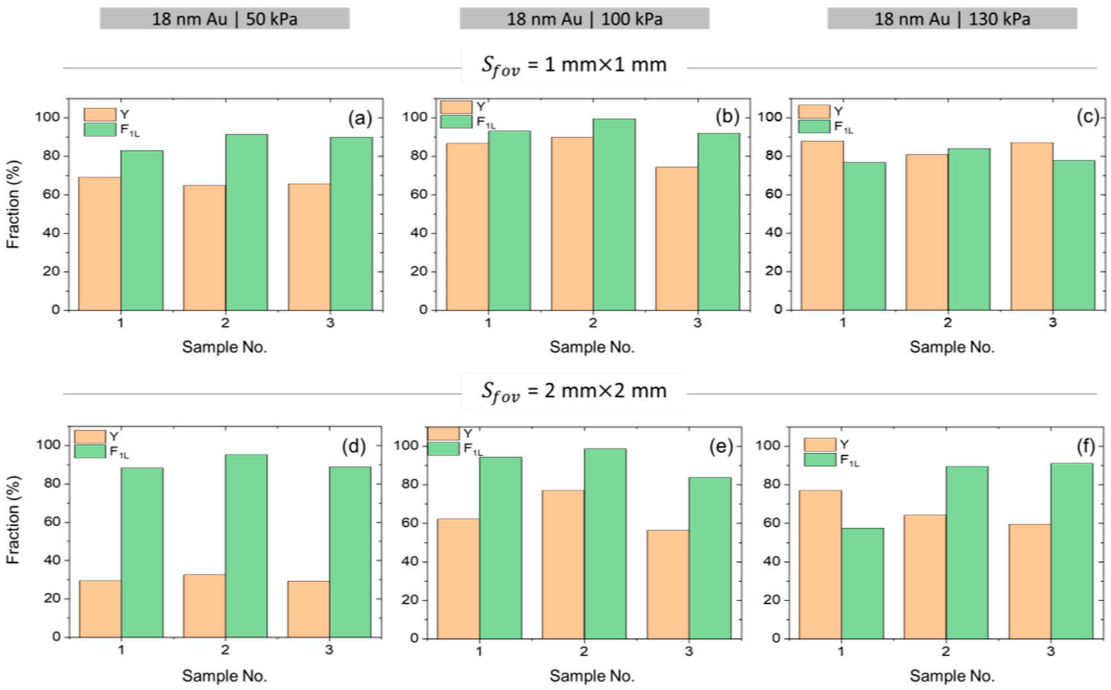

Supplementary Figure S3. Consistency check of the statistics of the exfoliation yield. Using 18 nm-thick Au films, three samples are fabricated at each pressure: 50 kPa, 100 kPa, and 130 kPa, totaling 9 samples. Optical microscope images are used to calculate the yield (Y) and monolayer fraction (F_{1L}) as presented in the main text. (a-c) Statistics using S_{fov} = 1 mm × 1 mm. At each pressure, the Y and F_{1L} for three different samples show a variance mostly below 10%. The maximum variance is 15.7% for the Y in (b), which could be a estimate of the uncertainty of Y and F_{1L} obtained through this statistical method. This uncertainty is sufficient to resolve the pressure dependence in Figure 2 with a change larger than 16%. (d-f) Statistics using S_{fov} = 2 mm × 2 mm with the same set of data. Since the three most covered regions (in separate locations) are used for statistics, it is expected that the Y would decrease when using a larger S_{fov}. Indeed, comparing the corresponding upper and lower panels, Y drops significantly. However, the relative relation of Y as a function of P remains unchanged; e.g., Y at 50 kPa is lower than at the other two pressures, both in (a-c) and in (d-f). Although the bulk MoS_2 on the tape appears to have a size of nearly 10 mm × 10 mm, the exfoliation yield across the entire 10 mm × 10 mm wafer is quite non-uniform, as seen in the photo in Fig. 1b, which indicates that millimeter-sized continuous 1L-MoS2 coexists with millimeter-sized bare Au areas. We tentatively attribute this non-uniformity to the quality of the bulk MoS_2. In this work, choosing S_{fov} = 1 mm × 1 mm enables us to focus on the best exfoliation conditions and helps exclude possible uncontrollable factors.

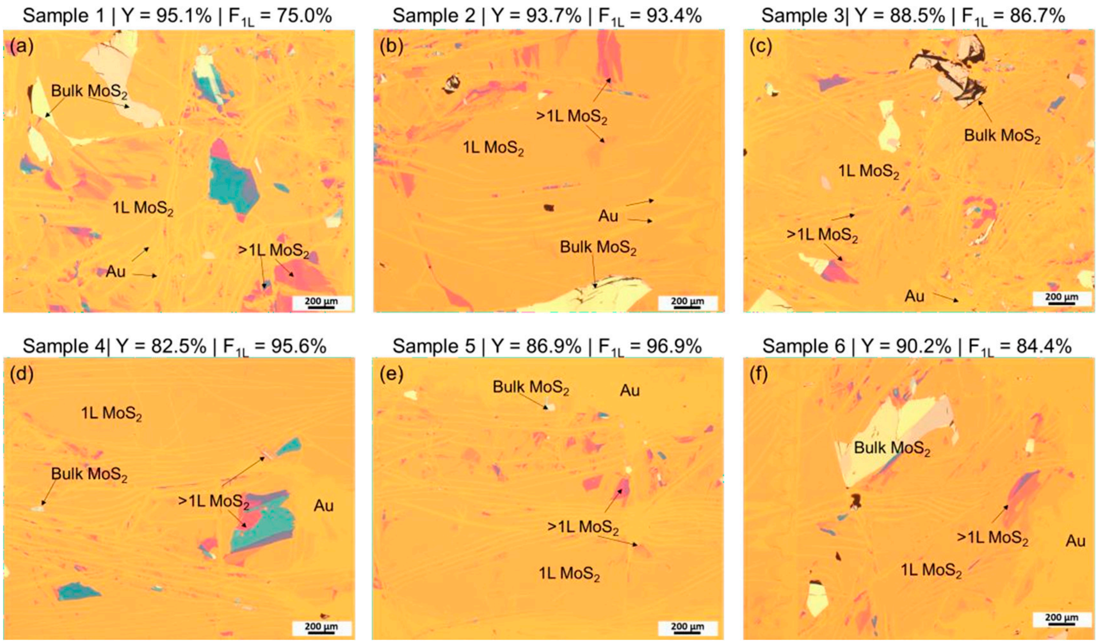

Supplementary Figure S4. Optical microscope images of six samples made with hand-applied pressures. The samples are made with 18 nm Au films. Pressure is applied through a soft cloth held by hand. The pressure is estimated by pressing the force gauge and is found to be within the range of 50 to 300 kPa. Sample index, yield (Y), and monolayer fraction (F_{1L}) are labeled at the top of each image. Surface structures, such as MoS_2 and uncovered Au surfaces, are indicated in the images.

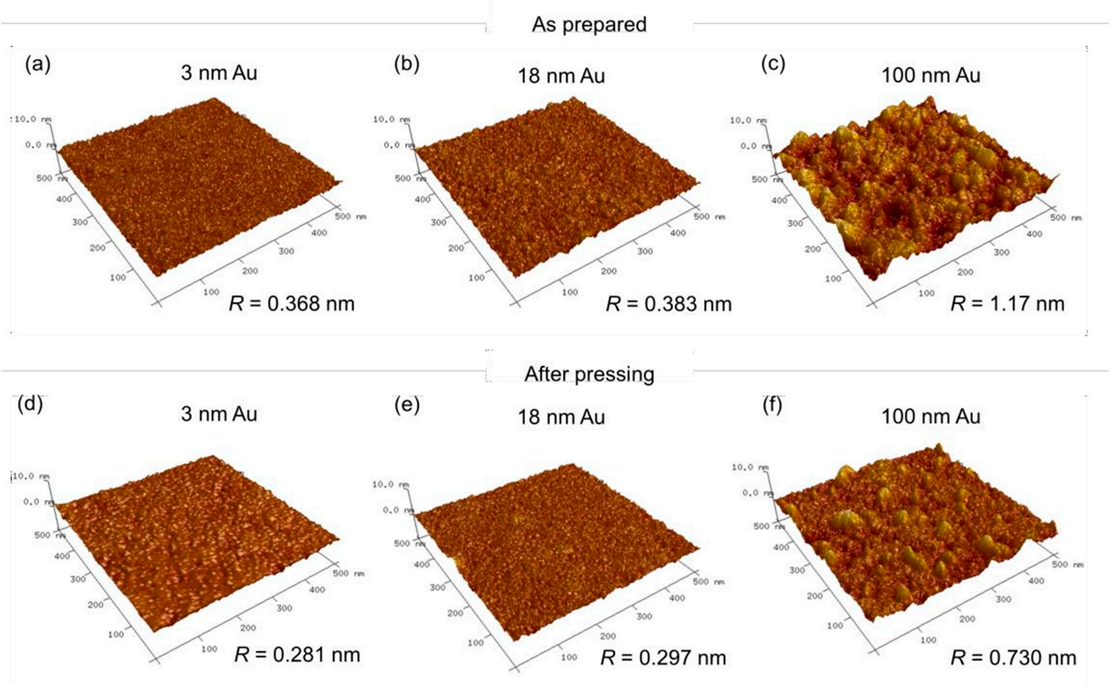

Supplementary Figure S5. Atomic force microscopy morphology of gold films before and after applying pressure. The topography is shown before (a–c) and after (d–f) applying 1000 kPa pressure to 3 nm, 18 nm, and 100 nm gold films, respectively. The post-pressure images are obtained from areas not covered by MoS_2 to avoid the influence of the MoS_2 layer on surface morphology. All images are presented with the same three-dimensional scale for easy comparison. The root mean square roughness (*R*) is indicated in each image.

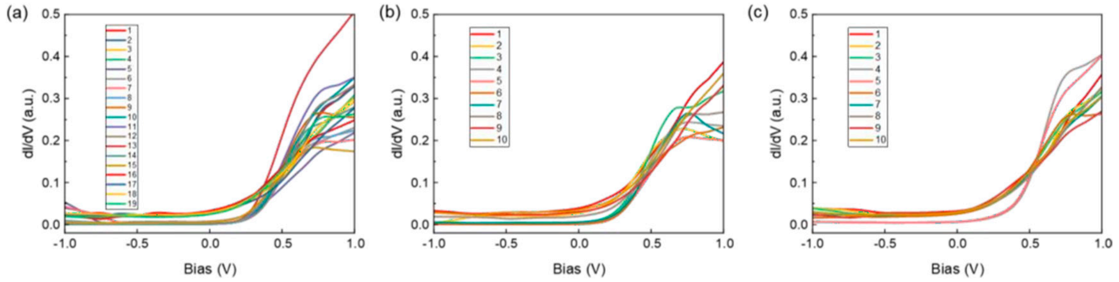

Supplementary Figure S6. d*I*/d*V* spectra of the imperfections on the 1L-MoS_2 covered Au surface. **P**anels (a), (b), and (c) show the spectra taken along a line across the "E", "F", and "G" structures in Figure 5e of the main text. The numbers in each plot correspond to the locations indicated in Fig. 5e, starting from the left.
